# Effectiveness of training on de-escalation of violence and management of aggressive behavior faced by health care providers in a public sector hospital of Karachi

**DOI:** 10.12669/pjms.342.14432

**Published:** 2018

**Authors:** Lubna Baig, Sana Tanzil, Shiraz Shaikh, Ibrahim Hashmi, Muhammad Arslan Khan, Maciej Polkowski

**Affiliations:** 1 Prof. Dr. Lubna Baig, MBBS, MPH, MMEd FCPS, PhD. APPNA Institute of Public Health, Jinnah Sind Medical University, Karachi, Pakistan; 2 Dr. Sana Tanzil, MBBS, FCPS. APPNA Institute of Public Health, Jinnah Sind Medical University, Karachi, Pakistan; 3 Dr. Shiraz Shaikh, MBBS, FCPS. APPNA Institute of Public Health, Jinnah Sind Medical University, Karachi, Pakistan; 4 Dr. Ibrahim Hashmi, MBBS. APPNA Institute of Public Health, Jinnah Sind Medical University, Karachi, Pakistan; 5 Dr. Muhammad Arslan Khan, MBBS. Aga Khan University, Karachi, Pakistan; 6 Mr. Maciej Polkowski, MA. International Committee of Red Cross, Islamabad, Pakistan

**Keywords:** De-escalation training and Health care provider, Violence

## Abstract

**Background & Objective:**

Considering high burden of violence against healthcare workers in Pakistan APPNA Institute of Public Health developed a training to prevent reactive violence among healthcare providers. The purpose of this training was to equip healthcare providers with skills essential to control aggressive behaviors and prevent verbal and non-verbal violence in workplace settings. This study assesses the effectiveness of training in prevention, de-escalation and management of violence in healthcare settings.

**Methods:**

A quasi-experimental study was conducted in October, 2016 using mixed method concurrent embedded design. The study assessed effectiveness of de-escalation trainings among health care providers working in emergency and gynecology and obstetrics departments of two teaching hospitals in Karachi. Quantitative assessment was done through structured interviews and qualitative through Focus Group Discussions. Healthcare providers` confidence in coping with patient aggression was also measured using a standard validated tool”.

**Results:**

The overall self-perceived mean score of Confidence in Coping with Patient Aggression Instrument “(CCPAI)” scale was significantly higher in intervention group (Mean= 27.49, SD=3.53) as compared to control group (Mean= 23.92, SD=4.52) (p<0.001). No statistically significant difference was observed between intervention and control groups with regard to frequency of violence faced by HCPs post training and major perpetrators of violence..

**Conclusion:**

De-escalation of violence training was effective in improving confidence of healthcare providers in coping with patient aggression.

## INTRODUCTION

Violence against Health Care Providers (HCPs) is a major problem in both developed and developing countries. Fear of violence affects the performance of HCPs and decreases their responsiveness to healthcare needs of the patients especially in emergency settings.^1^ Lack of security may also decrease the confidence of the patient in availing services from the hospitals.^2^ While the developed countries have made a significant progress in providing a safe work environment to their health care providers, violence against health care providers remains a significant public health problem in developing countries.^3^-^5^

Situation in Pakistan is dismal as hundreds of HCPs have been killed in the last decade as a result of terrorism, crime, sectarian divide and extremist elements in the society.^6^ Previous studies have reported that physical and verbal abuse of all kinds is frequently experienced by HCPs working in major public hospitals in Karachi, Pakistan.^7^,^8^ A recent multicentre research conducted in Karachi reported that around one third of all health care providers had experienced some kind of violence in the past 12 months.^9^ The study identified immediate need of effective interventions at various levels including training of HCP’s to equip them with essential communication skills, de-escalation of aggressive/violent behavior and management of Post Traumatic Stress Disorder (PTSD) as a result of violence.

Evidence from various parts of the world supports the effective role of trainings for de-escalation of violence for HCPs. These trainings helped in reducing impact and frequency of violence and improved patient-providers` interactions in healthcare settings.^10^-^13^ The ICRC and its team of public health experts developed a training manual for de-escalating aggressive behavior to prevent violence against healthcare providers.

The content of the four hours de-escalation training comprised of four modules which are:

1. Understanding Violence and Stress, (includes information from baseline study regarding burden and types of violence against healthcare providers and major reasons of violence in healthcare setting).
2. Escalation and De-escalation of violence (includes techniques of de-escalation of aggressive behavior using verbal and non-verbal techniques).
3. Management of Post-Traumatic Stress Disorder (includes strategies for managing PTSD).
4. Patient-Communication Protocol (includes techniques of active listening and empathic communication, and methods of breaking bad news in potentially violent situations).

Training included varied teaching methodologies including brainstorming, videos based on scenarios and role plays on doctor-patient interactions which may potentially cause reactive violence. Master trainers from AIPH conducted those trainings in the public sector tertiary care hospital of Karachi which were the intervention site.

This study aimed to assess the effectiveness of training for prevention and de-escalation of violence by HCP’s after four months. The ultimate aim was to scale-up this intervention if results showed better skills of the trained HCPs in de-escalation of violence.

## METHODS

This Quasi-experimental study using mixed methods Concurrent Embedded design was conducted in October, 2016. The study was conducted among health care providers currently working in Emergency, Gynecology & Obstetrics, Medicine & Allied and Surgery and Allied departments of two tertiary care teaching hospitals of Karachi. The assessment was conducted simultaneously after four months of de-escalation training at the intervention hospital and a control hospital of similar scale where trainings were not conducted.

The Quantitative assessment was done through a structured questionnaire. Study participants for intervention group were randomly selected from a list of 147 healthcare providers (HCPs) who had received des-escalation trainings at intervention hospital while controls were selected from the hospital where trainings were not conducted. The HCPs at intervention hospital that were not working at the time of data collection were excluded from the study. With an assumption of overall 20% reduction in the frequency of violence faced by all trained healthcare providers in intervention group as compared to control arm at 5% level of significance and a power of 80% the minimum sample size of 154 was obtained i.e. 77 in each group.

Participants from each study site were selected using non-probability convenience sampling technique. For control arm, healthcare providers from emergency and other departments who had been working in these settings for at least past four months were approached. Data was collected by trained data collectors using structured questionnaire to collect information regarding frequency, types and reasons of violence in control and intervention groups. Confidence levels of HCPs in dealing with agitated patients was measured using a tool adapted from “Confidence in Coping with Patient Aggression Instrument “(CCPAI)” scale.^14^

Data was analyzed using SPSS version 20. Descriptive statistics are reported as frequencies and percentages. The intervention and control group ware compared to identify possible differences in their demographics, frequency of violence experienced and aggregate CCPAI scale scores using Chi-Square Test for categorical variables and Independent T-test for quantitative variables.

For Qualitative study two Focus Group Discussions (FGDs) at each site (total of four FGDs) were conducted with doctors and nurses working in emergency and other relevant departments by authors of this paper. Each FGD was recorded and transcribed in Urdu and later translated into English. The data was analyzed using thematic content analysis. Both ‘manifest content’ (visible, obvious components) and ‘latent content’ (underlying meaning) of the text was analyzed.

## RESULTS

Quantitative data was collected from 141 HCPs including: 71 from intervention and 70 from control hospital. The FGDs included a total of 30 participants (14 in intervention and 16 in control arm).

The study participants in intervention and control hospitals were comparable as no statistically significant differences were found between them for socio-demographic variables. The departmental affiliations varied significantly (p ;< 0.05) (Table-I).

**Table-I:** Demographic and occupational characteristics of the study participants in Intervention and Control groups.

Variable	Intervention (n=71)	Control (n=70)	P value
Age Mean (SD)	27.34 (6.17)	29.86 (8.55)	0.173
***Gender***			
Male	25(35.2%)	24(34.3%)	0.908
Female	46(64.8%)	46(65.7%)	
***Current Position***			
Doctor	50(70.4%)	54(77.1%)	
Nurse	11(15.5%)	14(20.0%)	0.054
Medical Student	10(14.1)	2(2.9%)	
***Department***			
Emergency	21(29.6%)	19(27.1%)	
Gynecology &Obstetrics	8(11.3%)	6(8.6%)	
Medicine & Allied	10(14.1%)	34 (48.6%)	<0.001
Surgery & Allied	34 (48.6%)	11(15.7%)	
***Years of work experience***			
<1 yrs	39(54.9%)	32(45.7%)	
1-5 yrs	19(26.8%)	18(25.7%)	0.334
>5 yrs	13(18.3%)	20(28.6%)	

There were no statistically significant differences regarding frequency of experiencing or witnessing any kind of violence at work and types of major perpetrators of violence in the last four months.. The healthcare providers of the intervention hospital had higher average scores on CCPAI scale (Mean=27.49, SD=3.53) as compared to control (Mean=23.92, SD=4.52) (p<0.001) (Table-II).

**Table-II:** Frequency of violence experienced or witnessed by healthcare providers in intervention and control groups.

Variable	Intervention (n=71)	Control (n=70)	p-value
Experienced violences	17 (23.9%)	17 (24. 3%)	0.962
Witnessed violence	31 (43.7%)	36 (51.4%)	0.356
None	28 (39.4%)	27 (38.6%)	0.916
Number of Times violence was faced by those who experienced it: Mean (SD)	n=17	n=17	
	2.3 (1.16)	3.00 (1.83)	0.229
***Perpetrator***	n=43	n=43	
Attendant	42 (97.7%)	42 (97.7%)	1.00
Patient	1 (2. 3)	1 (2. 3)	

**Fig.1:**
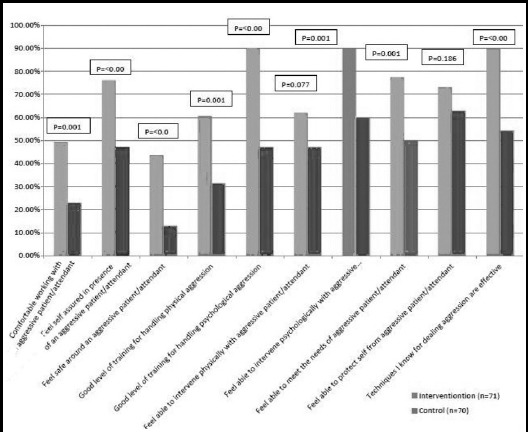
Comparison of individual items of CCPAI scale between intervention and control groups.

The proportion of self perceived confidence was significantly higher for eight out of ten items in intervention group that received training on de-escalation of violence as compared to control (Fig.1).

For qualitative data analysis coding of transcripts was done by two independent researchers and consensus was reached on three themes including recall of training content, positive experiences and recommendations.

HCPs in intervention group were able to recall contents from training modules mainly related to communication and de-escalation; however majority was not able to recall strategies for management of Post Traumatic Stress Disorder (PTSD). When asked about PTSD module, HCP’s at intervention hospital said, i) “Nobody has experienced this kind of disorder; perhaps we have not applied it that is why we have forgotten it”, ii) “We get over stress very quickly realizing that some form of violence is inevitable in our field.”

HCPs at the intervention group acknowledged that training helped them control their temperament in a challenging environment and also enabled them to effectively practice active listening and empathy. One of the HCP mentioned that, “I learnt how to respond to different behaviors of patients and maintain composure”. They also felt that their counseling practices have improved and they felt more confident communicating with patients and avoid lengthy altercation and possible violence.

The control group said that they lack confidence in dealing with aggression, one HCP said, “Running away is the best option”. HCPs in control group managed aggression and violence by applying self learnt strategies including counseling of patient, maintaining silence when abused, and agreeing with perpetrators to control violence. HCP’s, in control group strongly emphasized the need of training for coping with aggression and violence at work (Table-III). The training topics and needs identified by HCPs in control group were similar to the content covered through des-escalation trainings conducted.

**Table-III:** Perceptions and Practices of health care providers regarding management of violence.

	Intervention		Control	

	FGD1 Gynae	FGD 2 ER	FGD1 Gynae	FGD 4 ER
Recall of training Content	-How to control and balance temperament -How to respond to aggressive behavior -Right way to counsel patients	-How to calm down an aggressive patient -How to identifylikely violence and deal with it -Talk less and communicate with one person only -Listen carefully to patients/attendants and try to understand what they said	Not applicable	
Positive Experiences	-Dealt aggression by listening to patients/attendants and explaining the process of care -Explained the situation beforehand -Helped a patient in getting prompt care -Tried to calm down attendants and talk to immediate relative -Made patients understand the course of disease to help them cope with it -Didn’t react to anger: stayed quiet and then explain			
Training Needs	Not applicable		-How to communicate bad news -How to deal with aggressionw - How to counsel a patient/attendant -How to Communicate with senior staff	-Stepwise approach to breaking bad news -Dealing aggression -How to communicate the progress of serious patients -How to remain polite while interacting with angry attendants
Recommendations	-Should be mandatory before House Job and included in medical curriculum -Refreshers every 6 months-1year -Should be divided into two sessions -More scenarios should be added -Should be contextualized for different settings and different levels of people	-Should be included in Nursing Curricula -Refreshers should be done -Timing should be flexible	N/A	-Trainings should be given at undergraduate level -Trainings should comprise of real life scenarios -Training material should be realistic and correlate with the environment and type of situation -Duration should be two hours

### Suggestions from intervention and control group

1. The participants from both groups strongly recommended that de-escalation trainings must be institutionalized and included as part of medical and nursing curricula at undergraduate level.
2. The participants from both groups also recommended periodic refreshers for HCPs in practice.
3. Both groups suggested that training should be conducted in short sessions of one or two hours as attending a four hours are difficult to manage within their busy work schedules.
4. HCPs in both groups also emphasized the need of raising public awareness on respecting HCPs a doctor said: *“Definitely communication by healthcare provider matters a lot but equally important is societal behavior towards healthcare providers”*

## DISCUSSION

This is the first study of its kind in Pakistan which attempted to assess the effectiveness of trainings for de-escalating and managing violence in healthcare settings. We found that HCPs in intervention group had higher perceived confidence levels and coping skills to deal with aggression when compared with the control group. We did not find any statistically significant differences in the frequency of patient aggression faced by HCPs in intervention and control groups. These finding are consistent with findings reported by a systemic review published in 2015 which included studies published between January 2000 and September 2011. The systematic Review of 9 studies reported improved confidence levels and coping skills to deal with aggression among HCPs who received des-escalation of violence trainings but no change in frequency of patient aggression incidents.^12^ The reduction in the incidence of aggression and violence in healthcare settings requires multipronged strategies in addition to training for de-escalation of violence. These strategies should include improved secure working conditions for the HCPs, media awareness campaigns regarding respect for HCPs, legal protection to HCPs and above all increasing literacy level of general public.^15^-^17^

We found that HCPs in intervention group acknowledged that de-escalation training had improved their attitude and temperament towards aggression expressed by patients and their attendants. These findings are consistent with studies conducted by Grenyer and Collins.^18^,^19^ In the Grenyer study statistically significant increase was observed in understanding of aggression and violence management strategies among the HCPs’ after attending violence management training.^18^ This study also found increase in confidence.^18^ In the evaluation study by Collins` of Prevention and Management of Aggressive Behaviour Programme it was found that training had a positive effect on nurses attitude.^19^

Our results are also congruent with the study conducted in Stockholm, Sweden, which showed that violence prevention and management training can influence the HCPs attitude and can improve work place environment in healthcare settings.^20^

We found that HCPs who had received de-escalation of violence training could recall most of the training content except the training related to PTSD. This in our opinion could be due to the perceived usefulness of training and that they may have used some of the strategies suggested in the training for de-escalation of violence. This could also explain why PTSD training module was not recalled as it may not have been utilized as much.

The study participants suggested multipronged approaches to reduce incidence of violence in healthcare settings. HCPs from intervention and control groups suggested similar interventions including raising awareness regarding respect for HCPs, incorporation of violence prevention trainings in medical and nursing curricula at undergraduate level and refresher trainings for trained HCPs. These findings are in concordance with the studies done by Oostrom & Mierlo, and Lehmann et al.^10^,^21^

The study participants also recommended regular refresher courses to maintain learning for HCPs already in practice. These recommendations are consistent with the suggestion of HCPs in previously conducted studies.^22^-^24^

### Limitations of the Study

the scale of this study is limited to comparison of two tertiary care hospitals of Karachi which may not be a representative sample of all HCPs in Karachi.

## CONCLUSION

De-escalation training was found effective in improving confidence of healthcare providers in coping with patient aggression. There is a need to upscale and institutionalize de-escalation of violence trainings for HCPs.

### Ethical approval

Ethical approval for this study was obtained from Institutional Review Board (IRB) of Jinnah Sindh Medical University and Dow University of Health Sciences. Informed consent was obtained before each interview and confidentiality of participants was ensured.

### Author`s Contribution

**LB:** Conceived the idea, designed the study and the de-escalation of violence training manual, and did final edits on the manuscript.

**ST:** Participated in coding and analysis and wrote the first draft of manuscript.

**SS:** Participated in coding and statistical analysis, did the training on de-escalation of violence and wrote the methodology section of the manuscript.

**IH:** Participated in data collection, coding and data analysis, also wrote the introduction section of the manuscript.

**MAK:** Participated in data collection, training on de-escalation of violence, coding and wrote the results section.

**MP:** Participated in conceptualizing the project and edited the last draft.
